# Imatinib Alters Agonists-mediated Cytoskeletal Biomechanics in Lung Endothelium

**DOI:** 10.1038/s41598-017-14722-0

**Published:** 2017-10-26

**Authors:** X. Wang, R. Bleher, L. Wang, J. G. N. Garcia, S. M. Dudek, G. S. Shekhawat, V. P. Dravid

**Affiliations:** 1 Tianjin Key Laboratory of the Design and Intelligent Control of the Advanced Mechatronical System, Tianjin University of Technology, Tianjin, China 300384; 2 National Demonstration Center for Experimental Mechanical and Electrical Engineering Education, Tianjin University of Technology, Tianjin, China 300384; 3 Department of Materials Science and Engineering, Northwestern University, Evanston, IL USA 60208; 4 Department of Medicine, University of Illinois, Chicago, IL USA 60612; 5 Department of Medicine, University of Arizona, Tucson, AZ USA 85721

## Abstract

The endothelium serves as a size-selective barrier and tightly controls the fluid exchange from the circulation to the surrounding tissues. In this study, a multiplexed microscopy characterization is developed to study the spatio-temporal effects of Abl kinases on endothelial cytoskeletal structure using AFM, SEM, and immunofluorescence. Sphingosine 1-phosphate (S1P) produces significant endothelial barrier enhancement by means of peripheral actin rearrangement. However, Abl kinase inhibition by imatinib reduces rapid redistribution of the important cytoskeletal proteins to the periphery and their association with the cortical actin ring. Herein, it moderates the thickness of the cortical actin ring, and diminishes the increase in elastic modulus at the periphery and cytoplasm. These findings demonstrate that imatinib attenuates multiple cytoskeletal changes associated with S1P-mediated endothelial barrier enhancement and suggest a novel role for Abl kinases in mediating these S1P effects. These observations bridge the gap between molecule dynamics, structure complexity and function connectivity across varied length-scales to improve our understanding on human pulmonary endothelial barrier regulation. Moreover, our study suggests a framework for understanding form-function relationships in other biomechanical subsystems, wherein complex hierarchical organization programmed from the molecular scale to the cellular and tissue levels has an intimate relationship to the overall physiological function.

## Introduction

One of the most technically complicated features of living organisms is the ability of cells to sense mechanical forces and appropriately convert them into biological responses. The hierarchical organization programmed from the molecular scale to cell and tissue levels influences the abilities to sense, generate, and bear mechanical forces occurring across varied length-scales with diverse functional characteristics. The elegance of complexity in biological systems makes it exceedingly difficult to characterize the various components and depict their internal functional connectivity both spatially and temporally. Many prior studies have established important connections between structural characteristics, mechanical responses and physiological functions of cells, tissues and organs in order to better understand the fundamental mechanisms underlying multiple disease processes, including vascular diseases^1,2^, cancer^3–6^, cardiomyopathies^7,8^ and Alzheimer’s Dementia^9,10^. These studies provide a greater understanding of how mechanics relates to the biological functions of the body, which may lead to better diagnosis and treatment of many progressive illnesses.

The endothelium tightly controls the exchange of fluid from the circulation to the surrounding tissues. A significant and sustained increase in vascular permeability underlines the pathophysiology of multiple disorders that affect critically ill patients, such as acute respiratory distress syndrome (ARDS)^11^. Despite multiple clinical trials over the past few decades, targeted pharmacologic therapies are still lacking. The mortality rate is still as high as 30–50% in ARDS and severe sepsis^12^. The size-selective characteristics of the barrier to plasma proteins and other solutes is a key factor in maintaining fluid balance of tissue. In addition to the biochemical functions, these biological processes also embody a complex balance of mechanics. Actin filaments, a dynamic structural framework in the EC cytoskeleton, combine structural integrity and mechanical stability enabling network organization and structuring^13^. In our previous work, we investigated the correlation between mechanical properties and cytoskeletal structure of human pulmonary artery endothelial cells using *correlative* multiplexed microscopy, in response to the barrier-disrupting agent, thrombin, and the barrier-enhancing agent, sphingosine 1-phosphate (S1P), respectively^14^. Meanwhile, the dynamics, activity and stability of several critical kinases play an important role in actin filament rearrangement. The Abl family kinases, including c-Abl and Abl related gene (Arg), have well known roles in binding and bundling F-actin filaments, in phosphorylating cytoskeletal effector proteins, and in modulating the activity of non-muscle myosin light chain kinase (nmMLCK) and Rho family GTPases^15^. Therefore, the critical role of Abl kinases in endothelial barrier regulation has spurred recent interest in the potential for modulating their activity in the treatment of vascular barrier dysfunction. However, the physiological function of Abl kinases in actin filament structural arrangement, and its ultimate effect on cellular mechanical properties and barrier permeability, especially in response to barrier-enhancing agents such as S1P, have yet to be fully elucidated. This is a clinically important area necessary to develop medical therapies capable of attenuating vascular leak and resuming normal endothelial barrier function.

Imatinib is a small commercially available molecule inhibitor, blocking the ATPase activity of the kinase c-Abl, Abl-related gene (Arg), platelet-derived growth factor receptor (PDGFR), c-KIT, and discoid domain receptor-1^16^. Thus far, its pharmaceutical role has been used primarily in the treatment of chronic myeloid leukemia and gastro-intestinal stromal tumors clinically^16^. However, nonmalignant proliferative disorders such as lung fibrosis^17^ and pulmonary hypertension^18^ may represent future prospects for its additional clinical applications. In this current study, we compare the cellular mechanical responses of human lung endothelial cells in which Abl kinases have been inhibited by imatinib with non-inhibited cells. The comparison was performed in response to well-characterized barrier-enhancing agent, S1P, using atomic force microscopy (AFM) both spatially and temporally. Concurrently, the rearrangement of cytoskeletal structure is evaluated using scanning electron microscopy (SEM), and the relocation/colocalization of critical cytoskeletal effector proteins with cortical actin filaments is identified via immunofluorescence. The observations from both SEM imaging and immunofluorescent confocal microscopy serve as complementary techniques to interpret the cellular mechanical properties alteration based on AFM characterization. This multiplexed microscopy/characterization provides novel insights into how Abl kinases affect cellular cytoskeleton modulation, deciphering connectivity between complex hierarchical architecture and associated cellular/tissue response for the form-function relationship.

## Methods

### Reagents and antibodies

All reagents including sphingosine-1-phosphate (S1P) were purchased from Sigma-Aldrich unless otherwise specified. Imatinib was obtained commercially from LC Laboratories and reconstituted in DI water to yield a 20 mM stock. Dulbecco’s phosphate buffered saline (PBS) and trypsin were purchased from Life Technologies, while biological grade glutaraldehyde and paraformaldehyde used for cell fixation were obtained from Electron Microscopy Science. The following reagents and antibodies were acquired for immunofluorescence: mouse monoclonal anti-cortactin (p80/85) antibody clone 4F11 (EMD Millipore), mouse anti-paxillin antibody clone 349 (BD Biosciences), mouse anti-VE-cadherin antibody (Santa Cruz), Alex Fluor 488 goat anti-mouse IgG secondary antibody (Life Technologies), and Rhodamine-conjugated phalloidin (Molecular Probes).

### Cell culture

Human pulmonary artery endothelial cells were purchased from Lonza and cultured in manufacturer’s recommended complete endothelial growth medium-2-microvessel (EGM-2MV, Lonza) consisting of defined growth factors and supplemented with 10% fetal bovine serum (FBS, Sigma-Aldrich). Endothelial cells (EC) were maintained at 37 °C in a humidified incubator with 5% CO_2_ and utilized at passages 6–9.

### AFM characterization

Cells from each primary flask were detached with 0.05% trypsin, resuspended in fresh culture medium and transferred to 60 mm plastic petri dishes coated with collagen I (BD Biosciences) for AFM characterization. Both confluent (80–90% coverage of petri dish area) and sub-confluent cells (40–50% coverage) were used to ensure the presence of a monolayer or single cells as needed for AFM scanning. Before AFM imaging and measurements, cells were washed three times with sterile PBS to remove any debris that might stick to the AFM probe during the experiments. Complete growth medium was replaced with fresh medium supplemented with defined growth factors and 2% FBS to induce a basal state of the cells. AFM imaging and force measurements were performed on a 37 °C heating stage using a BioScope Catalyst atomic force microscope (AFM, Bruker) integrated onto an Axio Oberver.D1m (Carl Zeiss) inverted optical microscope. The system allowed precise lateral positioning of the silicon nitride AFM probe over the target cell.

ScanAsyst-Fluid probe (Bruker) with a nominal spring constant *k* ~ 0.7 Nm^−1^ and a nominal tip radius *R* ~20 nm was employed. The Peakforce mode in liquid was used to investigate the morphology alterations of Abl kinase-inhibited cells in response to the barrier-recovery agent S1P. To reduce stress on cells during AFM mechanical measurement, smooth curved colloidal probe with a diameter of ~840 nm SiO_2_ bead and ~30 nm gold coating on the backside of the silicon nitride triangular cantilever was used for elasticity measurements (Novascan Technologies) in force-volume mode. The deflection sensitivity was calibrated by repeated contact mode indentation on a clean glass slide (VWR International) in PBS and the spring constant of the compliant AFM cantilever was measured to be *k* = 0.12 ~ 0.15 Nm^−1^ by thermal noise method^19^. The tip radius was determined post-mortem by scanning electron microscope (SEM, Hitachi SU8030). The loading-unloading process was conducted at about 1.5 μm.s^−1^ and accomplished within roughly 1 second, during which the applied load, *F*, was measured as a function of the vertical actuation displacement of the piezoelectric cell, *y*, with 1024 data points collected. A maximum load of ~2 nN was applied at each data point, in order to keep the indentations on the cells within the elastic range^20^. During a typical experiment, a large scale (100 ~ 150 μm) contact mode AFM image was rapidly acquired at a resolution of 256 lines/frame, to locate an individual cell appropriate for measurements. A zoomed-in area was selected containing a part of a nucleus as well as periphery and cytoplasm in order to collect localized information of cellular mechanical properties. Measurements were carried out by acquiring arrays of 32 × 32 loading-unloading curves (force-volume map) within an area of 50 × 50 ~ 70 × 70 μm^2^ and each force-volume map was acquired over time periods of ~18 min. One force-volume map was first characterized on single EC grown on the coated petri dish before adding any stimulation. Then cells were challenged by sequentially adding solutions of 20 μM imatinib (54 min) for inhibiting Abl kinases, and 1 μM S1P (36 min) for stimulating barrier enhancement during data acquisition. Elasticity measurements were collected with three time-lapse force-volume measurements after imatinib treatment and another two after S1P stimulation on the same cell that lasted in total ~90 min (18 min × 5). Another sample, only treated with S1P (1 μM) as a control, was characterized with one time-lapse force-volume measurement before and another five frames after stimulation, which lasted in total ~90 min as well, in order to be consistent with the time used on the Abl kinase-inhibited sample. Five different cells were analyzed to generate the elastic modulus time-lapse response at each condition for the reproducibility.

The AFM cantilever connected to the piezoelectric cell, is used to deform the cell while the interaction force, *F*, is detected based on the deflection of the cantilever, δ_c_. The spring constant of the cantilever is *k* by the thermal noise method^19^. The raw AFM data is the relationship between force, *F*, and piezoelectric cell displacement, *y*, where *F* = *k* × δ_c_. Since the AFM cantilever is compliant, it is bending into the opposite direction, δ_c_, while the sample is indented by δ_s_. Thus, raw AFM force-displacement between (*F* vs. *y*) needs to be converted to the relation between applied force and cell deformation (*F* vs. δ_s_) by correcting cantilever deflection from the piezo displacement^14,21,22^. According to the spherical Hertzian contact mechanical model^23^, the constitutive relation for a rigid spherical probe with radius of *R*_AFM_ pressing vertically on an elastic half continuum with elastic modulus, *E*, and Poisson’s ratio, ν = 0.50, is used to compute the cell elastic modulus, given by

1 $$E=\frac{3}{4}(\frac{1-{\nu }^{2}}{{R}_{AFM}^{1/2}})\frac{\partial F}{\partial ({\delta }_{s}^{3/2})}$$

An in-house developed MATLAB (MathWorks, Inc.) code was used to obtain elasticity maps by analyzing all 1024 force curves in each force-volume mapping, where the fitting goodness values *R* ^2^ exceeded 0.85 in all curve fittings. Elastic property at each pixel was characterized by fitting of force-displacement curve to spherical Hertzian contact model. In this work, the fitting depth, δ_s_, is selected 100 nm ~ 200 nm for nucleus, 80 nm ~ 140 nm for cytoplasm and 20 nm ~ 40 nm for periphery. A line of zero force was defined from the average deflection of points on the force-displacement curve corresponding to the positions of the cantilever when it was far away from the surface.

### SEM imaging

The cytoskeleton has to be first uncovered for direct electron microscopy observation, and detergent lysis is the most common way to remove the cell membrane. EC was seeded to a 24-well plate containing a gelatin-coated (0.25% in PBS) 12-mm glass coverslip in each well and allowed to recover in complete medium overnight. Four coverslips were prepared simultaneously from the same batch and passage of cells. Before any stimulations, the cells were briefly rinsed with warm sterile PBS and rendered quiescent in growth medium containing 2% FBS for 3 hrs. One coverslip acted as a control, the other three were stimulated with either imatinib (20 μM) for 54 min, or S1P (1 μM) for 36 min, or imatinib (20 μM) for 54 min followed by S1P (1 μM) for 36 min, in the humidified incubator. After stimulation, the cells were immediately extracted with 500 μL buffer composed of 1% non-ionic detergent Triton X-100 and 4% PEG (MW 40,000) in stabilization buffer for 15 min, followed by stabilizing in 500 μL buffer containing 50 mM imidazole, 50 mM KCl, 0.5 mM MgCl_2_, and 0.1 mM EDTA (pH adjusted to 7.1 using concentrated HCl) for 15 min. The cells were rinsed with PBS and then fixed with 500 μL of fixation solution (2.5% glutaraldehyde, 2% paraformaldehyde and 0.5% tannic acid in PBS with final pH adjusted to 7.1) for 15 min, briefly rinsed three times with PBS and three times with DI. Coverslips were then placed in the coverslip holder, where they were dehydrated through a series of ascending ethanol concentrations of 25%, 50%, 75%, 90% and 100% for 5 min each, followed by critical point drying (Samdri-795, Tousimis). Finally, the actin filament fine structure was coated with 10 nm osmium (OPC60A, Filgen) and images were captured using a SEM (Hitachi SU8030) at an accelerating voltage of 1 kV.

### Immunofluorescence confocal microscopy

Indirect immunofluorescence of endogenous cortactin (critical cytoskeletal effector protein), paxillin (focal adhesion protein) and VE-cadherin (adherens junction protein) were assayed. After trypsinization, ECs were added to 35 mm μ-Dishes with grids (provided by ibidi GmbH) and allowed to recover in complete medium with 10% FBS overnight. Cells were washed three times with warm sterile PBS and rendered quiescent in growth medium with 2% FBS 3 hrs prior to any stimulation. Four samples were prepared simultaneously, where one was used as a control. The other three samples were challenged with either S1P (1 μM, 36 min), imatinib (20 μM, 54 min), or imatinib (20 μM, 54 min) followed by S1P (1 μM, 36 min) in the incubator. After stimulation, cells in the μ-Dishes were immediately fixed in 4% paraformaldehyde/PBS (pH 7.4) for 15 min and permeabilized with 0.1% Triton X-100 for 10 min. The unreacted aldehyde groups were quenched in 50 mM glycine/PBS (pH 7.4) for 15 min and the non-specific binding was blocked in 5% BSA/PBS for 30 min (pH 7.4). Incubation with primary antibody of interest (mouse anti-cortactin (1:500), mouse anti-paxillin (1:500), or mouse anti-VE-cadherin (1:250) in 5% BSA/PBS) were performed for 2 hrs at room temperature. Goat anti-mouse IgG (1:500 in 5% BSA/PBS) conjugated to Alex Fluor 488 was selected as secondary antibody for 1 hr incubation. Actin filaments were visualized by staining cells with rhodamine-conjugated phalloidin for 30 min followed by nuclei staining with DAPI for 5 min with protection from ambient light. Analysis of immunofluorescent staining was performed using an inverted laser-scanning confocal microscopy system (Zeiss Axio Observer.Z1) with a 40× oil objective lens. Eight-bit images were acquired sequentially by scanning line by line with resolution of 1024 × 1024 using Zen 2009 software. Cells were representative of changes observed in three different sets of experiments. All post-acquisition image processing and quantitative analysis were performed using NIH ImageJ and Adobe Photoshop. The quantitative analysis was performed on *n* = 20 images at each condition.

## Results and Analysis

### Cellular morphology characterized by AFM

**Figure 1:**
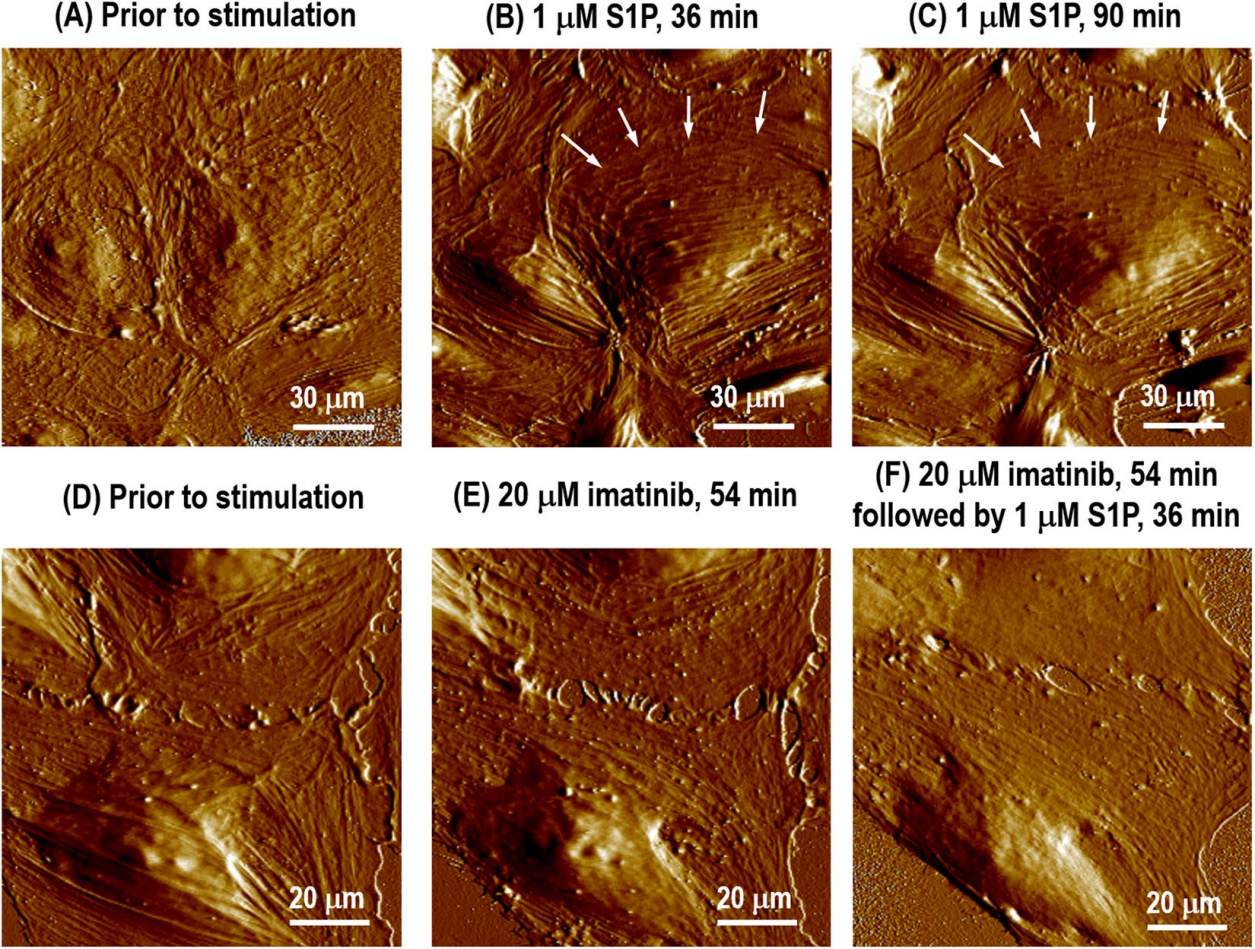
AFM scanning images from Peakforce error channel showing monolayer live human lung endothelial cells without any apparent intercellular gaps prior to any stimulation (**A**); stimulated with S1P (1 µM) showing the enhanced formation of the cortical actin ring (**B**, 36 min; **C**, 90 min). To investigate the biological function of Abl kinases on S1P-induced cytoskeleton rearrangement, monolayer live cells AFM scanning images from Peakforce error channel were obtained prior to any stimulation (**D**); 54 min after treatment with 20 μM of the Abl kinase inhibitor imatinib (**E**); and 54 min after treatment with 20 μM imatinib followed by 36 min treated with 1 μM S1P (**F**). Arrows in (**B**) and (**C**) indicate the cortical ring.

To link the critical biological function of Abl kinases^15^ with cellular morphology changes in response to S1P, live EC monolayer or single cells were analyzed with serial high-resolution AFM imaging under a physiologically viable fluidic specimen stage. This was performed after the sequential addition of 20 µM imatinib for 54 min to inhibit Abl kinases, followed by 1 µM S1P for 36 min to stimulate barrier enhancement. 20 µM imatinib is a clinically relevant concentration that was found to be optimal in prior dose-dependent studies and has no effect on VE-cadherin distribution/expression on static EC^24^, while 1 µM S1P has been found to produce rapid and dramatic enhancement of polymerized F-actin and myosin light chain phosphorylation at the cell periphery^25,26^. The treating times for both imatinib and S1P are adopted to get the maximum agonist effects^24,26^. Endothelial barrier function is dependent on a complex balance between the intracellular contractile forces generated by actin-myosin contraction, and cellular adhesive/resistive forces produced by cell-cell/cell-matrix interactions and rigid cytoskeletal components (microtubules and intermediate filaments)^27,28^. Figure 1 demonstrates live monolayer morphology alteration of Abl kinase-inhibited EC (imatinib treatment) in response to S1P compared to control EC by PeakForce mode scanning in liquid. Monolayer pulmonary ECs grown on collagen I coated plastic petri dishes display the polygonal shape in cobblestone appearance with intact borders and no apparent intercellular gaps as shown in Fig. 1A. S1P, a phospholipid generated by hydrolysis of membrane lipids in activated platelets and other cells, rapidly increases the level of cortactin and myosin at the cell edges, aiding in cell tethering forces and cell spreading, and finally enhancing EC barrier function^29,30^. 36 min after the addition of S1P, the cortical actin ring is well formed along the cell periphery, and strong interaction between adjacent cells is built through cell-cell interaction as indicated in Fig. 1B. A previous study revealed a rapid, Rac-dependent translocation of cortactin to the expanded cortical actin band following S1P challenge occurs within 5 min^31^. Therefore, the cortactn actin band is already well-established after 36 min treatment with S1P, and the S1P barrier-enhancing effect appears stable over time since there is no obvious difference between 36 min and 90 min stimulation (Fig. 1C). For the cells pretreated with the Abl kinase inhibitor imatinib, 54 min exposure to imatinib alone had minimal effects on cellular morphology changes in static EC, although several small intercellular gaps are formed that may be due to the inhibition (Fig. 1D–E). However, after a subsequent 36-min treatment with S1P, no substantial cortical actin or other apparent enhancement in cytoskeleton density occurs along the cell periphery (Fig. 1F), which is in contrast to the rearrangements that occur after S1P in the non-inhibited cells (Fig. 1C). These images demonstrate that imatinib inhibits important peripheral structural changes that occur in response to barrier-promoting agent S1P.

The changes in endothelial barrier function of a confluent monolayer within blood vessels are a consequence of integrated alterations in cytoskeletal structure at the level of each individual cell. However, examining changes in cell monolayers is complicated *in vitro* because of complex cell-cell interaction and hard-to-discern cell boundaries. Therefore, we next analyzed individual cells as a simplified system that allows each individual cell to function as its own control, in order to advance our understanding of the correlation between kinase biological function and cytoskeletal structure modulation.

**Figure 2:**
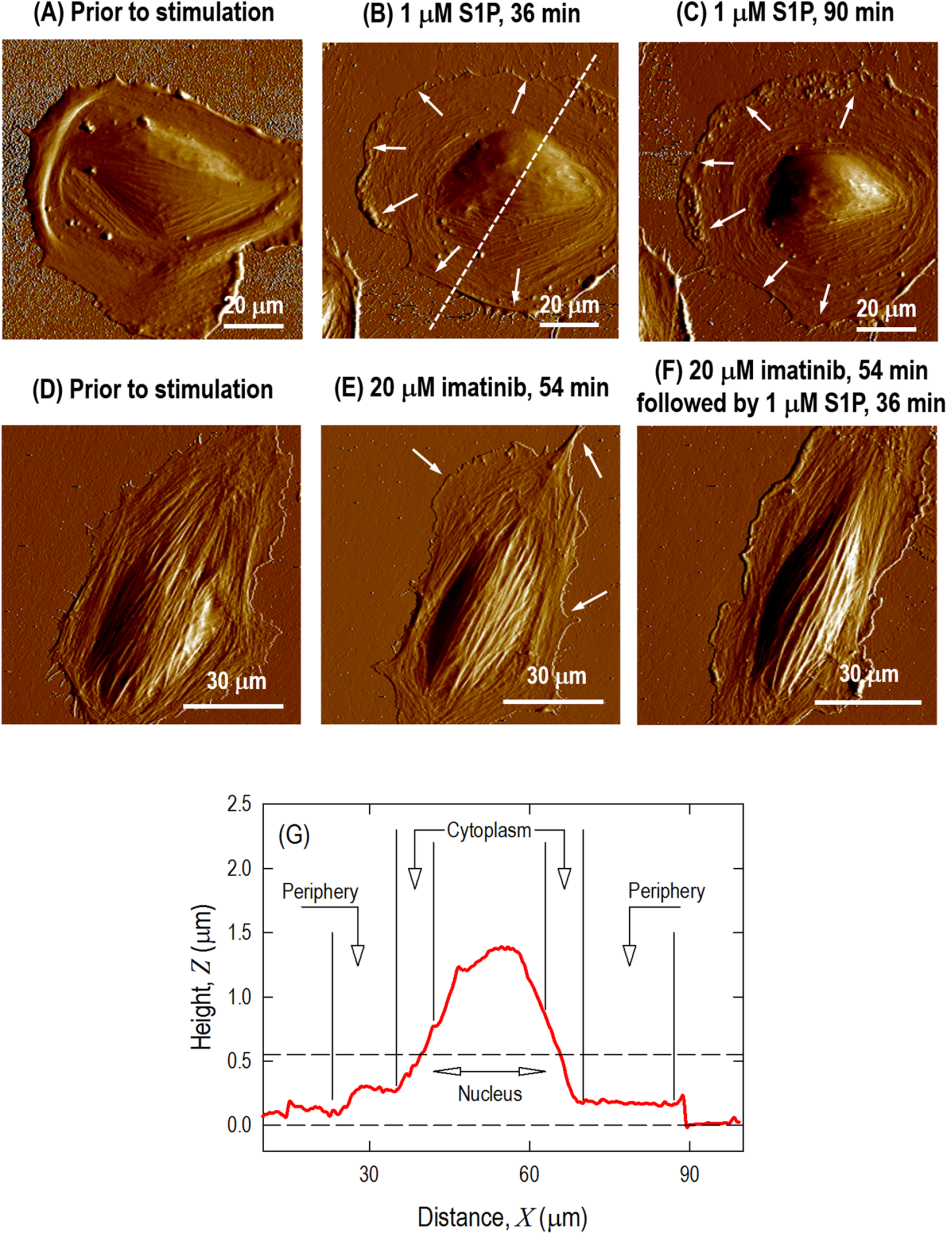
AFM morphology from Peakforce error channel of a single pulmonary EC grown on a plastic petri dish with no stimulation, showing actin filaments are randomly distributed with few peripheral bundles (**A**); the same cell is shown after S1P (1 µM) stimulation, which induces spreading of the cell periphery on the substrate and the formation of the cortical actin ring along the cell periphery (**B**, 36 min; **C**, 90 min). Another single live cell is shown prior to any stimulation (**D**); 54 min after treatment with 20 μM Abl kinase inhibitor imatinib (**E**) and 54 min after treatment with 20 μM imatinib followed by 36 min after treatment with 1 μM S1P (**F**). Cross-section height and distance profiles of the live cell in Fig. 2B showing the selection of 3 different regions of the cell labeled as nucleus, cytoplasm and periphery (**G**).

Without cell-cell interaction to obscure observation, the S1P effects are more prominent on the single cell level. Figure 2A demonstrates the profile characteristics of an individual pulmonary EC. The EC is well spread on the substrate and the cytoplasm contains active fibers with random distribution. Actin filaments are distributed along the edge of the cells forming low peripheral bundles, and fewer stress fibers are running across the top surface of the nucleus. Exposure of ECs to S1P (1 µM, 36–90 min) produces rapid translocation of multiple cytoskeletal proteins resulting in cell spreading and increased cortical actin as shown in Fig. 2B–C. This cortical actin accordingly assists in exerting tethering forces that inhibit cell contraction, aids cell spreading, and finally promotes endothelium barrier enhancement and recovery. Since the thick cortical actin band developed at the periphery, the cell is further spread upon the surface with an approximately round shape. Moreover, the stress fibers across the top of nucleus are reduced after S1P stimulation. However, the pre-inhibition of Abl kinases with imatinib substantially modulates the cell fate upon S1P exposure. With similar cytoskeleton structure, the cell displays an oval-shape prior to Abl kinases inhibition as shown in Fig. 2D. Little morphology change is induced after 54 min treated with imatinib as shown in Fig. 2E, although there is a cell leading edge retraction, which may due to imatinib treatment. Compared to the wide cortical actin band shown in Fig. 2B and C, pre-inhibition with imatinib weakens the S1P-mediated EC barrier enhancement effect represented by the absence of a typical cortical actin band forming along the periphery. The cell displays even higher aspect ratio, and the actin stress fibers across the top surface of nucleus are still apparent even after 36 min treated with S1P. These observations strongly suggest an important role of Abl kinases in the induction of endothelial cytoskeletal changes after S1P that are associated with the enhancement of endothelium barrier function (Fig. 2F). Because of live cells moving around in the image frame over which the AFM scanning is performed, the cellular morphology may be slightly different temporally, like cell-cell crosstalk shown in Fig. 2C and F. The cross-section profile of Fig. 2B is shown in Fig. 2G with selection of three different regions labeled as nucleus, cytoplasm and periphery. Baseline height of lung ECs were based on the measurements of 10 live cells: nuclear region 2.17 ± 0.3 µm, cytoplasmic region 0.56 ± 0.05 µm, and peripheral region 0.20 ± 0.05 µm. There is no apparent localized height alteration on the same cells after challenge with the various agents.

### Force mapping of stimulated ECs

**Figure 3:**
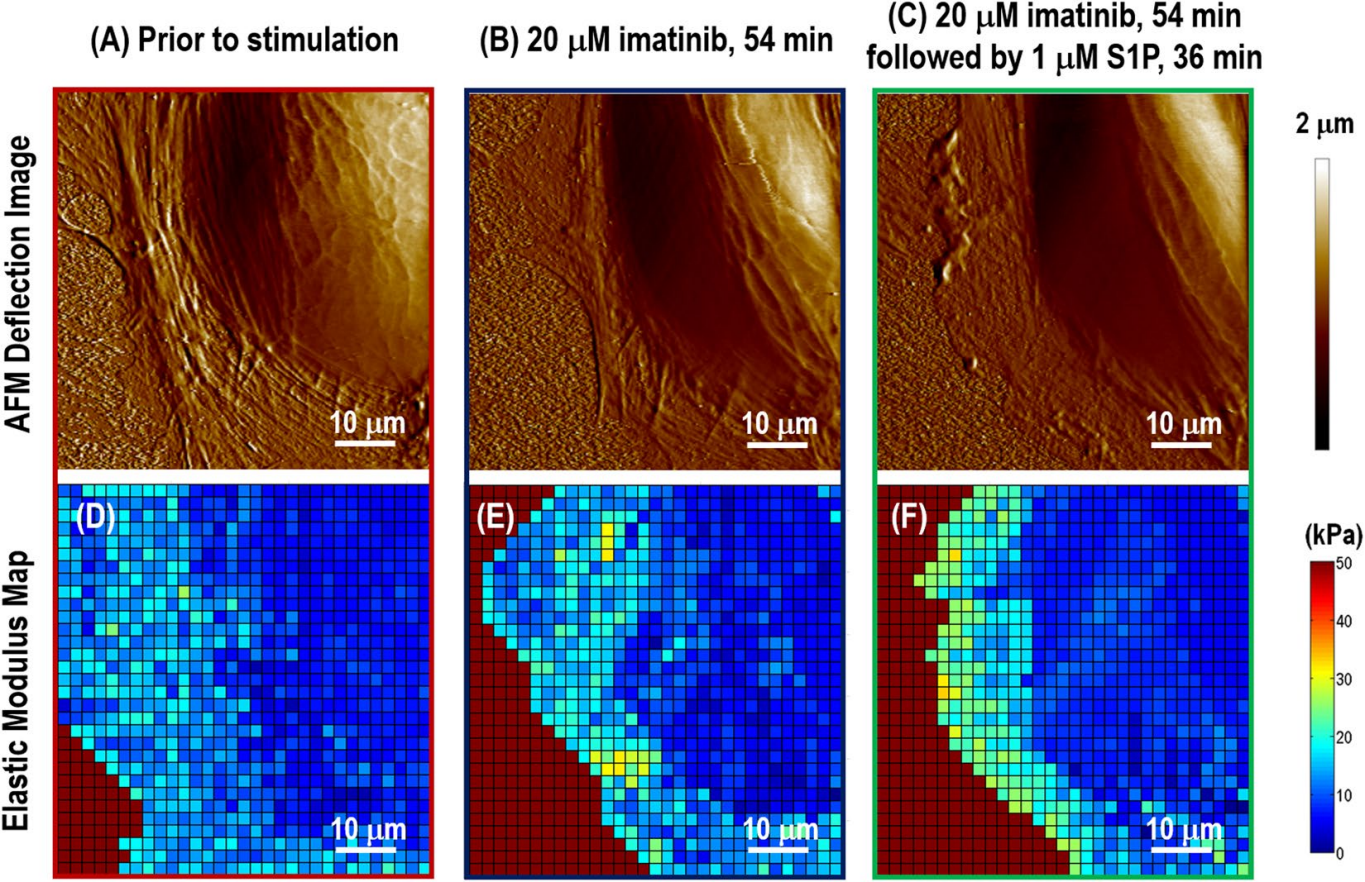
AFM deflection images of live EC prior to any stimulation (**A**); in response to 54 min after treatment with 20 μM imatinib (**B**) followed by 36 min treatment with 1 μM S1P (**C**). The mechanical measurements were carried out by acquiring arrays of 32 × 32 loading-unloading curves in the force-volume map. Elasticity at each pixel was characterized by variable-indentation-depth curve fitting of force-displacement curve to spherical Hertzian contact model using an in-house MATLAB code. The time-lapse elastic modulus maps prior to any stimulation (**D**); in response to 54 min after treatment with 20 μM imatinib (**E**); followed by 36 min after treatment with 1 μM S1P (**F**). Each pixel indicates the localized sub-cellular elastic modulus.

The elasticity distribution in live ECs can be dynamically determined using AFM force-volume mapping. To study the effects of Abl kinases on the changes in cellular mechanical properties when exposed to the barrier-enhancing agent, S1P, sequential AFM force-volume mappings on an individual cell were carried out at baseline before any stimulation, during 54 min after imatinib treatment, and during the following 36 min after S1P stimulation. To compare the cellular response under external load from the AFM probe, control cells were also characterized before and after S1P treatment within a 90 min period. Data at the cell periphery, cytoplasm and nucleus regions were collected in order to correlate localized biomechanical properties with detailed information on cytoskeletal structure gathered from SEM and protein redistribution acquired based on immunofluorescence, respectively. Figure 3 shows the AFM deflection images and time-lapse elastic modulus maps of a live EC prior to stimulation (A, D), 54 min after treatment with 20 µM imatinib (B, E), and followed by 36 min treatment with barrier enhancing 1 µM S1P (C, F).

**Figure 4:**
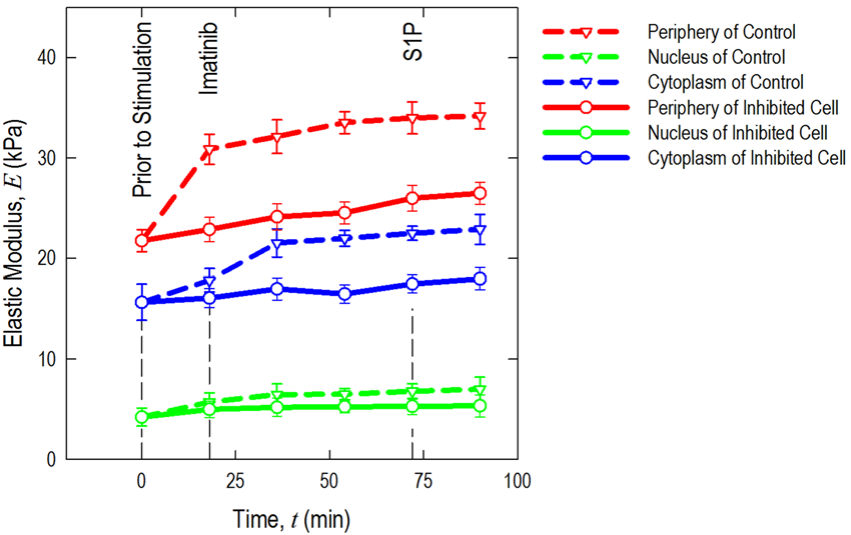
Quantification of live cell sub-cellular (periphery, nucleus, and cytoplasm) elastic modulus as a function of time in response to sequential 20 μM Abl kinases inhibitor imatinib (54 min) and 1 μM S1P (36 min) (as shown in solid line) compared with the same cell at baseline prior to stimulation. For comparison, another cell sample only treated with 1 μM S1P is used as a control (dashed line). *n* = 5 different cells are analyzed to generate elastic modulus time-lapse responses.

The cellular morphology is characterized using contact mode AFM scanning with the colloidal probe (colloidal probe diameter Φ ~ 1 μm) and resolution of 256 lines/frame. AFM indentation with resolution of 32 × 32 within in a 50 ~ 70 μm^2^ is performed after switching over to force-volume mode, and elastic modulus maps are generated by curve fitting to the spherical Hertz contact mode. Quantitative sub-cellular elastic modulus information from this mechanical characterization can be pooled and summarized as shown in Fig. 4. The solid lines indicate the mechanical properties of Abl kinase-inhibited cells, while dashed lines describe those of control EC without imatinib. In control cells, the mechanical properties begin to change within the first frame of AFM mechanical characterization after S1P treatment (~18 min) and are more prominent after ~36 min stimulation. The elastic modulus increases significantly by ~30% (*p* < 0.005, determined by two-tailed unpaired Student’s *t*-test) along the cellular periphery and ~20% (*p* < 0.005) increase in the cytoplasm in control. The increase on sub-cellular mechanical properties correlates with peripheral cortical actin that stabilizes cell-cell junctions, and results in the enhancement of endothelial barrier in a monolayer. This highly sensitive measurement on live human pulmonary ECs is consistent with the model of EC barrier regulation as we have previously reported^14^. The S1P-induced mechanical properties remain stable after ~36 min challenge. However, these S1P-induced increases are significantly attenuated in Abl kinase-inhibited ECs, with only a ~5% (no statistical meaning) increase at the periphery and a ~8% (no statistical meaning) raise in the cytoplasm. These results indicate that Abl kinase inhibition with imatinib in live human lung EC alters the biomechanical changes induced by S1P that mediate barrier enhancement.

### Cytoskeletal frame structure observation under SEM

The cytoskeleton gives the cell shape and allows for adaptation to the environment under biochemical stimulation and mechanical resistance to deformation when bearing an external load. The localized assembly and arrangement of the cytoskeletal architecture drive the machinery of cellular morphology changes. Visual techniques such as light and electron microscopy are critical tools for analysis of cellular structures and function in cell biology. SEM provides an intuitive means to reveal non-labeled cytoskeleton with structural details and makes it possible to correlate cytoskeletal pattern changes in various regions with mechanical properties measurements obtained from AFM.

**Figure 5:**
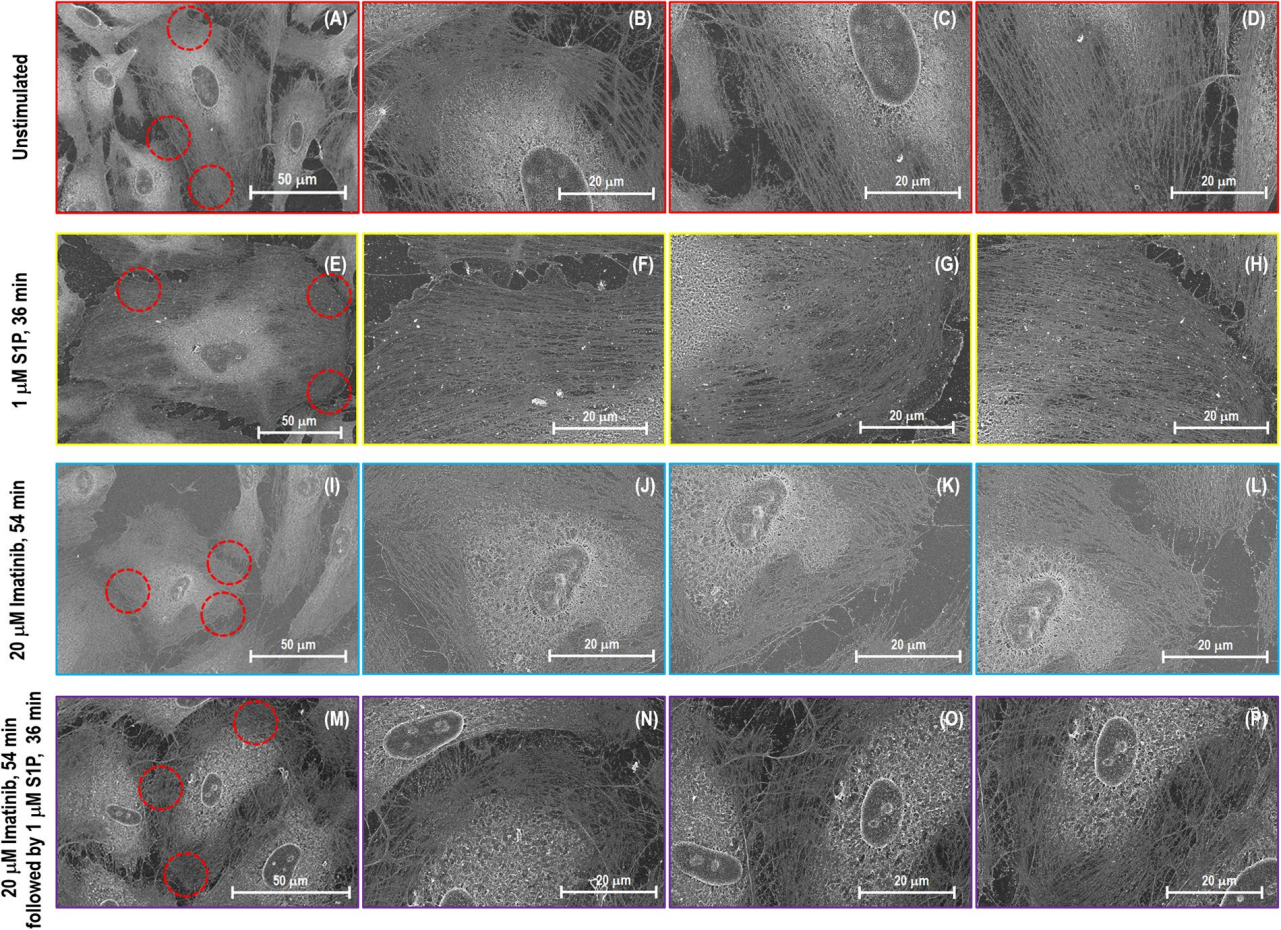
SEM images of actin filament structures of a single lung EC. Image (**A–D**), (**E–H**), (**I–L**) and (**M–P**) correspond to no stimulation, S1P alone (1 µM, 36 min), imatinib alone (20 μM, 54 min) and combination of imatinib (20 μM, 54 min) and S1P challenged (1 µM, 36 min) cells, respectively. (**B–D**), (**F–H**), (**J–L**) and (**N–P**) are the zoomed SEM images indicating the fine structure of actin filaments in the circles with red dotted lines on (**A**), (**E**), (**I**) and (**M**).

Figure 5 shows SEM images of human lung EC cytoskeleton structures after extracting the cell membrane. The sample has a 10 nm osmium coating since it is a non-conductive biological cell. The SEM images of the unstimulated cells demonstrate that the cobblestone morphology is exhibited with few active stress fibers distributed in the cytoplasm, and a low density of fibers scatted in the periphery with random orientation (Fig. 5A–D). In contrast, after stimulation with S1P (Fig. 5E–H), ECs show membrane protrusions with a high concentration of actin fibers at the cell periphery. The cell peripheral region reveals formation of a dense cortical actin band, which correlates to an increase in elastic modulus of the cell peripheral regions revealed by AFM. Peripheral extensions such as filopodia and lamellipodia are shown in the image, and the overall cellular size appears larger than observed at the other conditions (Fig. 5A–D, Fig. 5I–L and Fig. 5M–P). Although Abl kinase inhibition with imatinib induces no obvious cytoskeleton structural differences compared with the unstimulated cells (Fig. 5I–L), the combination of imatinib and S1P (Fig. 5M–P) results in a substantial loss of the critical dense actin cytoskeleton at the periphery of cells. This correlates with significant attenuation of the elastic modulus increase at the cellular periphery as shown from AFM force mapping (Figs 3 and 4). Abl kinase inhibition substantially decreases the S1P-induced cytoskeleton redistribution at the cellular periphery, demonstrating an integral role for Abl kinases in regulating baseline EC permeability and response to barrier-altering agonists.

### Immunofluorescence on cortactin, paxillin and VE-cadherin

**Figure 6:**
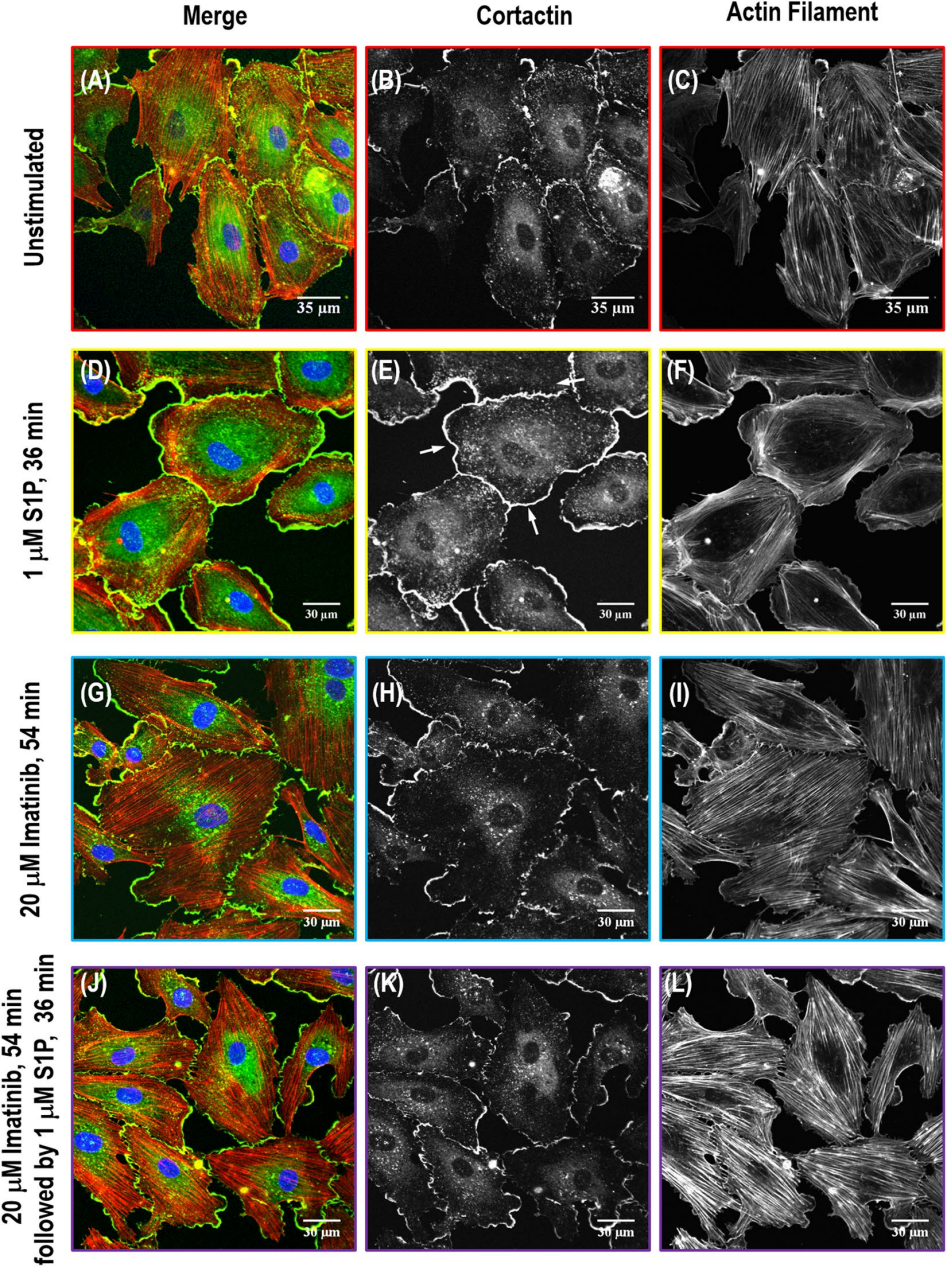
Immunofluorescence images of actin filament structures with cortactin distribution in human lung EC. Images are as follows: no stimulation (**A–C**), S1P alone (1 µM, 36 min) (**D–F**), imatinib alone (20 μM, 54 min) (**G**–**I**) and combination of imatinib (20 μM, 54 min) and S1P challenged (1 µM, 36 min) cells (**J–L**). (**A**, **D**, **G** and **J**) are merged images between actin filament (red) and cortactin (green). (**B,E,H,K**) are cortactin distribution pattern taken with Alexa 488 fluorophore and (**C,F,I,L**) are actin cytoskeleton distribution images with rhodamine-conjugated phalloidin.

**Figure 7:**
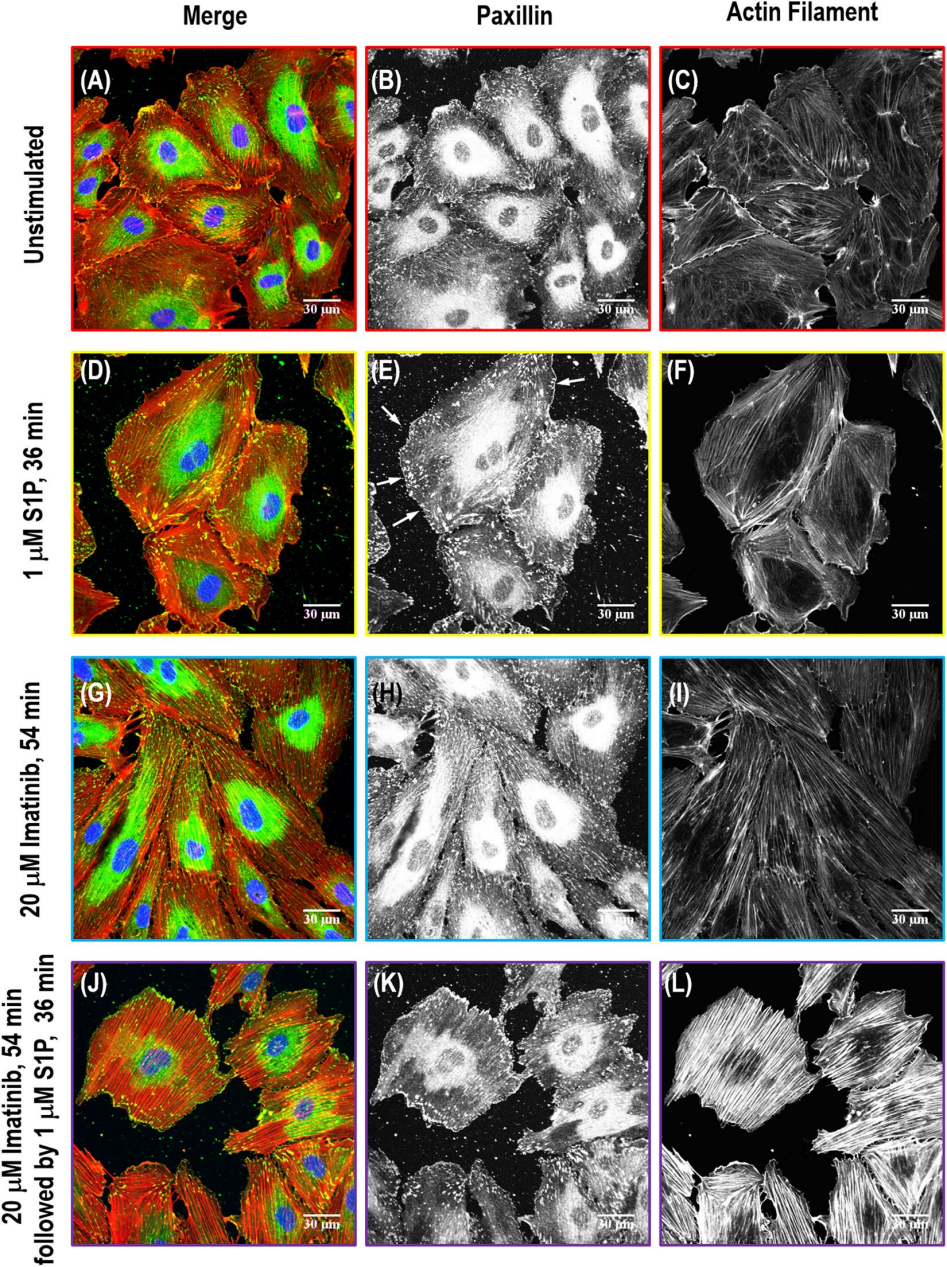
Immunofluorescence images of actin filament structures with focal adhesion paxillin in human lung EC. Images are as follows: no stimulation (**A–C**), S1P alone (1 µM, 36 min) (**D–F**), imatinib alone (20 μM, 54 min) (**G–I**) and combination of imatinib (20 μM, 54 min) and S1P challenged (1 µM, 36 min) cells (**J–L**). (**A**, **D**, **G** and **J**) are merged images between actin filament (red) and paxillin (green). (**B,E,H,K**) are focal adhesion paxillin distribution pattern taken with Alexa 488 fluorophore and (**C,F,I,L**) are actin cytoskeleton distribution with rhodamine-conjugated phalloidin.

**Figure 8:**
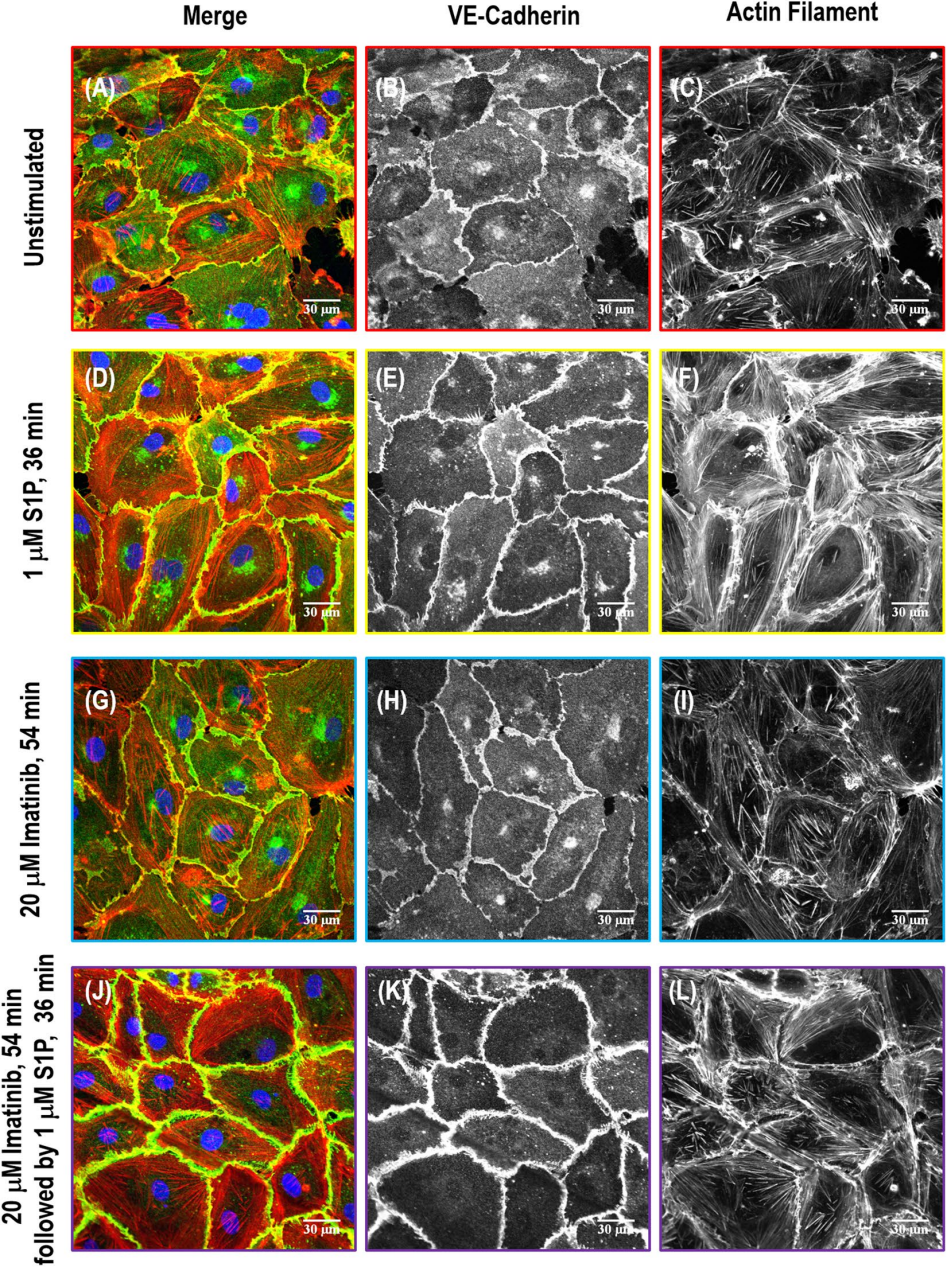
Immunofluorescence images of actin filament structures with VE-cadherin in human lung EC. Images are as follows: no stimulation (**A–C**), S1P alone (1 µM, 36 min) (**D–F**), imatinib alone (20 μM, 54 min) (**G–I**) and combination of imatinib (20 μM, 54 min) and S1P challenged (1 µM, 36 min) cells (**J–L**). (**A**, **D**, **G** and **J**) are merged images between actin filament (red) and VE-cadherin (green). (**B**,**E**,**H**,**K**) are adherens junction VE-cadherin distribution pattern taken with Alexa 488 fluorophore and (**C**,**F**,**I**,**L**) are actin cytoskeleton distribution with rhodamine-conjugated phalloidin.

Immunofluorescence serves as an informative approach to observe specific distribution of fluorescence-labeled protein in three-dimension with multi-color channels in order to reveal the dynamic assembly of cellular frame. To provide additional insights into the biological function of Abl kinases on actin cytoskeleton structural rearrangement, the relocation and colocalization of the critical proteins cortactin, focal adhesion protein paxillin and adherens junction protein VE-cadherin with the actin cytoskeleton structure were explored as shown in Figs 6, 7 and 8. Actin filaments were labeled with red rhodamine-phalloidin, and blue DAPI was used as a counterstain for nucleus identification. Meanwhile, the specific protein of interest for individual experiments (cortactin, paxillin, or VE-cadherin) was visualized by mouse antibodies staining followed by a secondary antibody conjugated to Alex Fluor 488.

**Figure 9:**
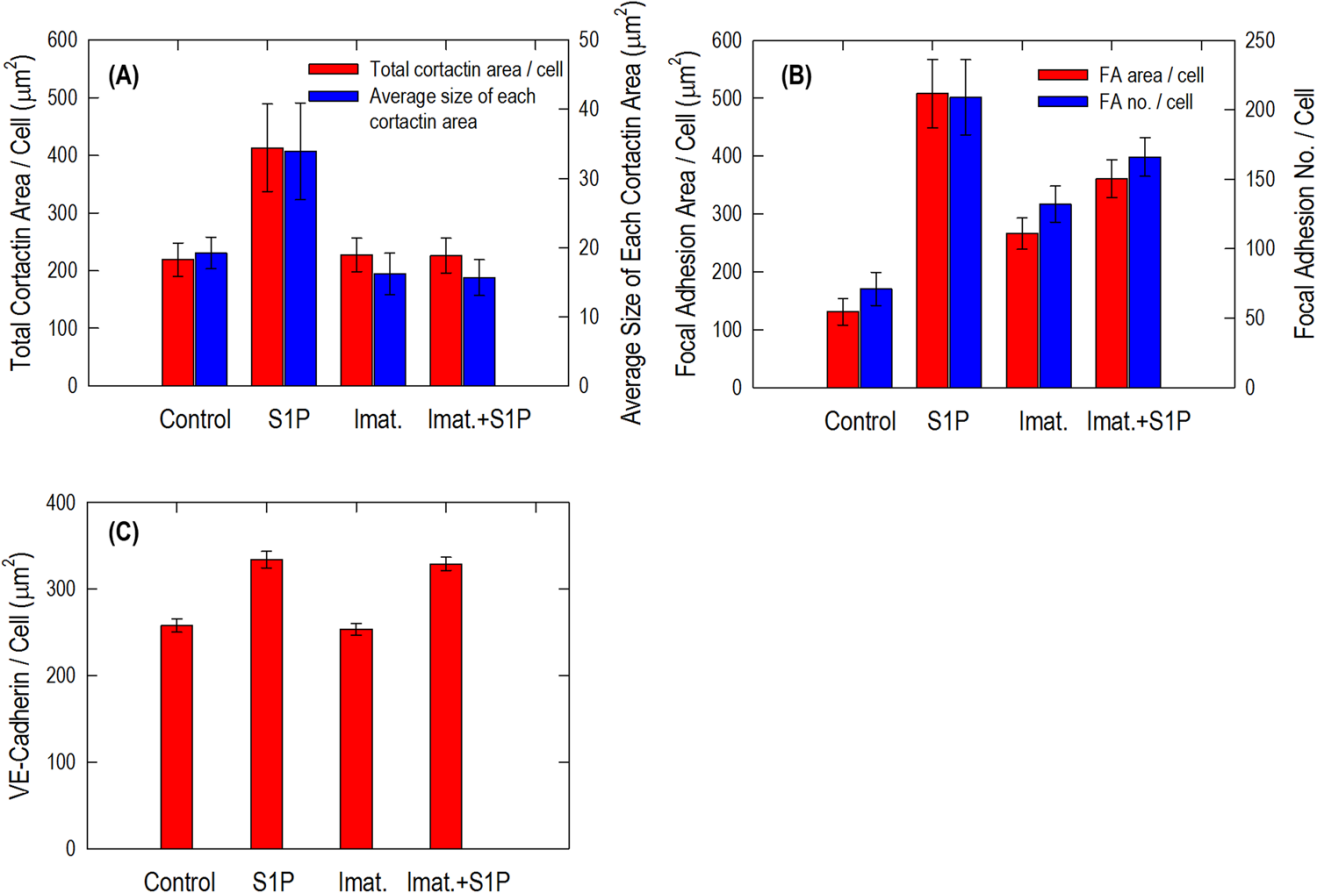
Quantitative analysis of total cortactin area/cell and average size of each cortactin area (**A**), focal adhesion area/cell and focal adhesion number/cell (**B**), VE-cadherin area/cell (**C**) in EC with no stimulation, S1P alone (1 µM, 36 min), imatinib alone (20 μM, 54 min) and combination of imatinib (20 μM, 54 min) and S1P (1 µM, 36 min). The quantitative analysis was performed on *n* = 20 images at each condition and the combination effect of imatinib and S1P significantly diminishes the S1P only-induced enhancement on both parameters (*p* < 0.005, determined by two-tailed unpaired Student’s *t*-test).

Cortactin (molecular weight ~80 kDa) is a monomeric protein located in the cytoplasm of cells that can be activated by external stimuli to promote polymerization and rearrangement of the actin cytoskeleton, especially the actin cortex around the cellular periphery^32^. Confocal fluorescent microscopy with indirect immunofluorescence reveals scattered and thin peripheral and cytoplasmic cortactin staining in the unstimulated human lung EC (Fig. 6A–C). After S1P, extensive translocation of cortactin to the cell periphery occurs as previously reported^31^. This rapid redistribution of cortactin to the EC periphery after S1P corresponds to membrane ruffling regions and colocalization with the cytoskeletal actin band (Fig. 6D–F). It demonstrates the important role of cortactin in promoting the phosphorylation of the peripheral myosin light chain (MLC), associated formation of actin band formation, lamellipodia and filopodia, and even cell migration, which is integral in cytoskeletal changes associated with EC barrier enhancement^31^. Imatinib alone has little effect on cortactin distribution at the cellular periphery since almost the same scattered pattern is observed as in the unstimulated cells (Fig. 6G–I). However, pre-inhibition of Abl kinases substantially blocks formation of the thick cortical actin band around the cellular periphery usually observed after exposed to barrier recovery stimulus, S1P (comparing Fig. 6K and E). After quantitative analysis on immunofluorescence channels, Fig. 9A shows pooled data indicating that S1P significantly increases total cortactin area per cell as well as the average size of each cortactin area. However, the combination effect of imatinib and S1P diminishes S1P-induced cortactin redistribution at the cellular periphery and results in the same cortactin area size as in the unstimulated cells. These studies suggest that Abl kinase inhibition remarkably attenuates S1P-induced cortactin rearrangements, supporting a critical role for Abl kinases in mediating cortactin structural formation and associated cytoskeletal rearrangements, which finally regulates EC barrier function.

Paxillin (molecular weight ~68 kDa) serves as an important docking protein at focal adhesions to recruit signaling molecules to a specific cellular compartment. It binds specific combinations of signaling molecules into a complex to coordinate downstream signaling^33^. The biological function of paxillin coordinated signaling is to regulate cell spreading and motility^34^. In unstimulated human lung EC, paxillin occurs in a dot-like pattern that is mostly diffuse in the cytoplasm at sites of stress fibers attachment, suggesting localization at focal adhesions as shown in Fig. 7A–C. After S1P stimulation, paxillin staining is more intense with longer and thicker patches and more localized at the cellular periphery. Merged images of actin filament and paxillin staining further demonstrate paxillin colocalizes with the peripheral actin ring, suggesting S1P recruits paxillin along the cortical band (Fig. 7D–F). Imatinib alone appears to increase the numbers of small focal adhesions (indicated by paxillin staining) compared with non-stimulated cells (Fig. 7G–I). However, the combination of imatinib and S1P significantly attenuates the increase in focal adhesion area and focal adhesion number based on quantitative analysis using ImageJ compared to S1P-only-treated cells (Fig. 7J–L). Abl kinase inhibition promotes paxillin focal adhesion formation as shown in Fig. 9B, although the specific signaling pathway needs further investigation. Moreover, there is no apparent cortical actin band ring forming after the treatment with both Abl kinase inhibitor, imatinib, and the barrier-enhancing agent, S1P, which is consistent with the cytoskeleton structure revealed by SEM imaging (Fig. 5).

Integrity of intercellular junctions is a major determinant of permeability of the endothelium. The VE-cadherin-based adherens junction is particularly important since VE-cadherin is known to be required for maintaining a restrictive endothelial barrier in cultured cells^35^. In the untreated control human lung EC, the expression of VE-cadherin is concentrated in inter-endothelial junctions with a thin and scattered pattern, showing partial continuity of adherens junctions between two cells (Fig. 8A–C). S1P induces accumulation of F-actin at the cell periphery and enhancement of continuous VE-cadherin-positive peripheral staining of ECs in the monolayer (Fig. 8D–F). Imatinib alone has no obvious effect on VE-cadherin distribution/expression alteration on EC, demonstrating almost the same pattern as the non-stimulated cells (Fig. 8G–I), which is consistent with the results^24^. However, Abl kinase inhibition with imatinib fails to alter S1P-induced enhancement of peripheral VE-cadherin (Fig. 8J–L), which is quantified in Fig. 9C, suggesting that Abl kinases at 20 μM does not regulate adherens junction remodeling under these conditions.

## Discussion

The EC cytoskeleton is a dynamic, functionally complex and spatially targeted contractile structure which is responsible for cellular responses to external stimuli, bioactive agonists, and mechanical stresses. EC challenged with the endogenous barrier-promoting compound S1P results in the recruitment of Abl kinases, phosphorylated nmMLCK and cortactin, rapid increases in Abl kinases activity, and spatial targeting of Abl kinases to barrier-promoting cortical actin structures^36^. Abl kinases phosphorylate nmMLCK and cortactin to enhance endothelial barrier function, demonstrating the central role of Abl kinases in actin cytoskeletal rearrangement^15^. Although the target effectors in EC responsible for these varied effects are poorly characterized, the interactions between Abl kinases, cortactin, and nmMLCK may be one of the key signaling events affecting lamellipodial formation, cytoskeletal remodeling, and focal adhesion dynamics to regulate EC barrier permeability.

In the current study, localized mechanical measurements from AFM (Figs 3 and 4), actin filament structures observation from SEM (Fig. 5), and protein relocation from immunofluorescence (Figs 6, 7, 8 and 9) were utilized to demonstrate the central role of Abl kinases in regulating cytoskeletal structure and cellular biomechanical properties. After S1P stimulation, the actin cytoskeleton is enhanced at the cell edges, seen as membrane ruffles and cortical rings, which increases the elastic modulus at the periphery and cytoplasm. However, pretreatment with the Abl kinase inhibitor imatinib attenuates S1P-mediated structural changes in human lung EC, reflected by reduced cortical ring formation, altered the increase of elastic modulus along the periphery, and decreased peripheral relocation of cortactin and paxillin protein. By combining immunofluorescence with SEM, it is possible to bridge the divide between the systemic distribution of molecules/proteins of interest and corresponding cytoskeleton structural complexity in order to address the integrated feedback loop between biomechanical elements and mechanical properties from AFM characterization.

One limitation of our data is that imatinib blocks the ATPase activity of several kinases including c-Abl, Arg, platelet-derived growth factor receptor (PDGFR), c-KIT, and discoid domain receptor-1^16^. Although the effects of imatinib on specific kinase activity is not characterized in the current study, the expression of Abl kinases is essential for mediating S1P-induced cortical actin formation, nmMLCK and cortactin tyrosine phosphorylation, and endothelial barrier enhancement^36^, which argues strongly for the interpretation that imatinib is mediating its effects on the S1P response primarily through the Abl kinases and their modulation of nmMLCK and cortactin, among other important cytoskeletal targets^37^. Using atomic force microscopy to correlate biophysical properties, cytoskeletal structural changes from SEM and specific protein relocation from immunofluorescence, there is a strong indication that cell remodeling is led by actin fiber rearrangement, which is induced by uneven distribution of critical protein inside the cell. These rearrangements/relocations result in cellular stiffness redistribution at cytoplasm and periphery. Herein, this novel study is conducted to aim at addressing the gap between molecule dynamics, structure complexity and function connectivity. Moreover, it provides a framework for understanding the form-function relationship in other biomechanical sub-systems.

## Electronic supplementary material

Supplementary Material for Imatinib Alters Agonists-mediated Cytoskeletal Biomechanics in Lung Endothelium
