# Supplementary material for: Imatinib Alters Agonists-mediated Cytoskeletal Biomechanics in Lung Endothelium
Source: Sci Rep. 2017 Oct 26;7:14152. doi: 10.1038/s41598-017-14722-0 (PMC5658337; doi:10.1038/s41598-017-14722-0)
Supplement: Supplementary file 1 — Supplementary Material for Imatinib Alters Agonists-mediated Cytoskeletal Biomechanics in Lung Endothelium [file 41598_2017_14722_MOESM1_ESM.pdf]

Supplementary Material for
Imatinib Alters Agonists-mediated Cytoskeletal Biomechanics in Lung Endothelium

X. Wang[1, 2, 3], R. Bleher[3], L. Wang[4], J. G. N. Garcia[5], S. M. Dudek[4*], G. S. Shekhawat[3*], V. P. Dravid[3*]

[1] Tianjin Key Laboratory of the Design and Intelligent Control of the Advanced Mechatronical System, Tianjin University of Technology, Tianjin, China. 300384

[2] National Demonstration Center for Experimental Mechanical and Electrical Engineering Education, Tianjin University of Technology, Tianjin, China. 300384

[3] Department of Materials Science and Engineering, Northwestern University, Evanston, IL, USA. 60208

[4] Department of Medicine, University of Illinois, Chicago, IL, USA. 60612

[5] Department of Medicine, University of Arizona, Tucson, AZ, USA. 85721

[*] Correspondence to:

sdudek@uic.edu, g-shekhawat@northwestern.edu, and v-dravid@northwestern.edu

Supplementary Materials:

Data analysis

In the force-volume mode, height information is also collected while performing indentation at each pixel within the scan size. The in-house developed MATLAB code read the height information from the height channel and used certain range at each pixel as fitting depth to fit the curve for elastic modulus as shown in Figure S1A. The interaction force, F, indents the cell with deformation of δ_s. According to the spherical Hertzian contact mechanical model (1), the constitutive relation for a rigid spherical probe with radius of R_{AFM} pressing vertically on an elastic half continuum with elastic modulus, E, and Poisson's ratio, $\nu = 0.50$, is used to compute the cell elastic modulus. Figure S1B is the representative raw AFM force-displacement curves collected at cell nucleus, cytoplasm and periphery. Figure S1C and Figure S1D are the curve fitting to spherical Hertz contact mechanics model for localized elastic modulus in log-log and linear plot.

According to the spherical Hertzian contact mechanical model (2), the constitutive relation for a rigid spherical probe with radius of R_{AFM} pressing vertically on an elastic half continuum with elastic modulus, *E*, and Poisson's ratio, $\nu = 0.50$, is used to compute the cell elastic modulus, given by

$$F = (\frac{4}{3} \times \frac{E}{1-\nu^2} \times \sqrt{R_{\mathrm{AFM}}}) \times \delta_s^{3/2} \qquad \text{(Eqn S1)}$$

In the log-log plot, it shows

$$\mathrm{Log}_{10}[F] = \mathrm{Log}_{10}[\frac{4}{3} \times \frac{E}{1-\nu^2} \times \sqrt{R_{\mathrm{AFM}}}] + \frac{3}{2}\mathrm{Log}_{10}[\delta_s] \qquad \text{(Eqn S2)}$$

Therefore, we can use the linear fitting with fix slope of 1.5 to get intercept for elastic modulus, *E*, in the plot of $\mathrm{Log}_{10}[F]$ vs. $\mathrm{Log}_{10}[\delta_s]$ as shown in Figure S1C. Actually, there are more than 10 cells tested per condition. During a typical experiment, a large scale (100 ~ 150 μm) contact mode AFM image was rapidly acquired at a resolution of 256 lines/frame, to locate an individual cell appropriate for measurements. A zoomed-in area was selected containing a part of a nucleus as well as periphery and cytoplasm in order to collect localized information of cellular mechanical properties. To maintain the resolution, the scan size decreases to ~ 50 × 50 μm^2. Since cell may move out of the frame during ~1.5 hrs treatment or cell may be dead during the scanning, thus, only 5 of cells whose results are close to the mean are used to summarize the time-lapse elastic modulus maps. Ten cyclic indentations were performed on cellular nucleus at the same position in order to verify the indentation was within elastic range as shown Figure S2, which shows our measurements are very consistent. There is no obvious plastic deformation as expected in cyclic indentation measurement. Therefore, a maximum load of ~2nN can keep the indentations on cells within the elastic range. Figure S3 shows the dependence of elastic modulus, *E*, on fitting depth, δ_s, with spherical probe on cell nucleus, which indicates *E*, as calculated using Eqn.S1, decreased with increasing δ_s. It reaches a plateau with increasing indentation. In this work, the fitting depth, δ_s, is selected 100 nm ~ 200 nm for nucleus, 80 nm ~ 140 nm for cytoplasm and 20 nm ~ 40 nm for periphery. The fitting depth is 400 nm ~ 500 nm with 5 μm spherical probe on normal and cancerous human cervical epithelial cell (3), 650 nm ~ 850 nm with 5 μm spherical probe on primary breast epithelial cancer cell (4), 100 nm ~ 300 nm with 4.5 μm spherical probe on HUVEC (5), which are comparable to ranges chosen in this work.

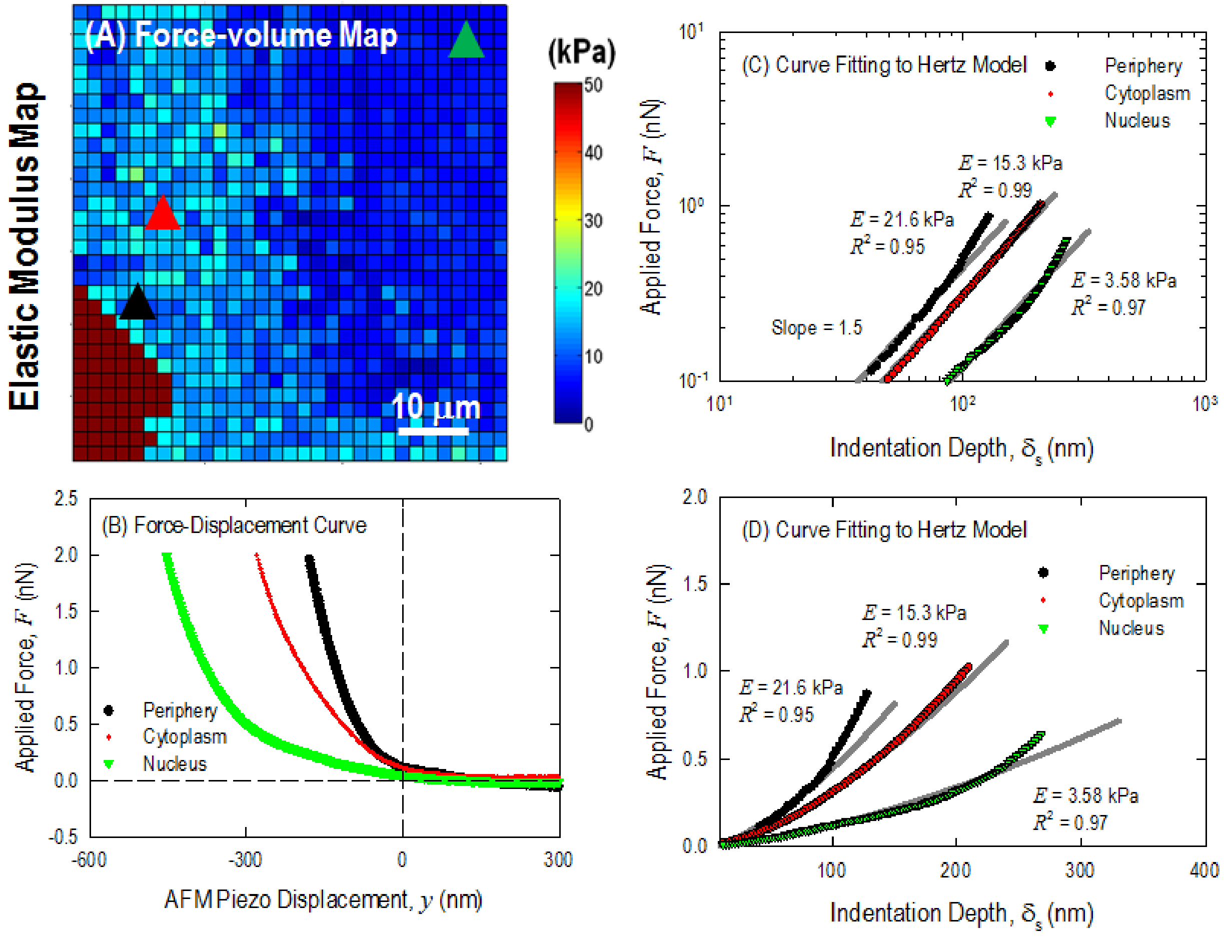

Figure S1. Curve fitting to spherical Hertz contact model at different regions for localized elastic modulus. (A) Elastic modulus map obtained from curve fitting. (B) The representative raw loading curves collected at the different regions of cells. (C) The curve fittings to spherical Hertz mechanical model for elastic modulus in the log-log plot (R^2 > 0.85). (D) The curve fittings to spherical Hertz mechanical model for elastic modulus in the linear plot (R^2 > 0.85)

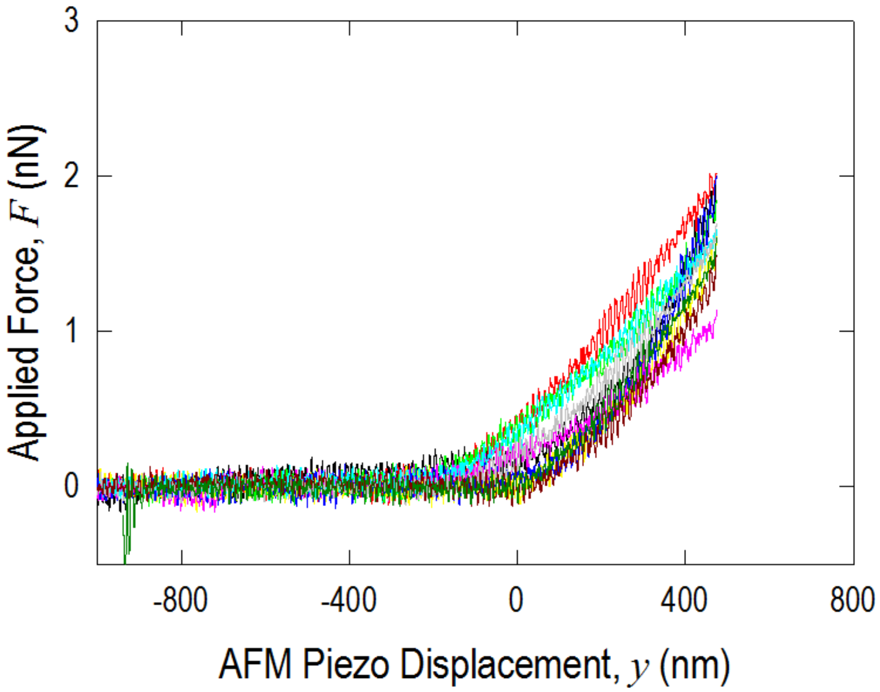

Figure S2. Ten cyclic indentation on cellular nucleus at the same position.

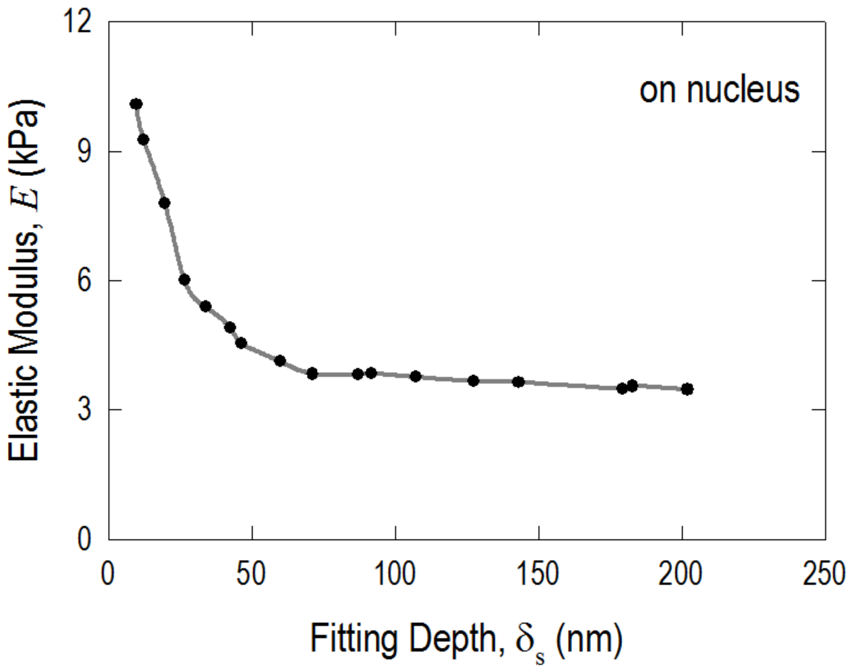

Figure S3. Sample of force-indentation curve measured on nucleus with spherical probe. Corresponding values of elastic modulus was dependent of fitting depth, δ_s.

Nanomechanics measurement on endothelial cells without any agents' treatment

In order to verify the morphology change and cytoskeleton rearrangement due to the imatinib blocking and S1P stimulation, nanomechanics measurements are first performed on endothelial cells without any agents' treatment. There is a heating stage combined with AFM to keep the measurement at physiological temperature. Furthermore, a plastic lid is used to cover the cell-culturing petri dish with a central hole designed for AFM scanner head while performing measurements, in order to keep the cells at good condition. Thus, the cell is quite stable. Figure S4 is two sets of images showing there is no obvious cellular morphology changes on non-stimulated endothelial cells.

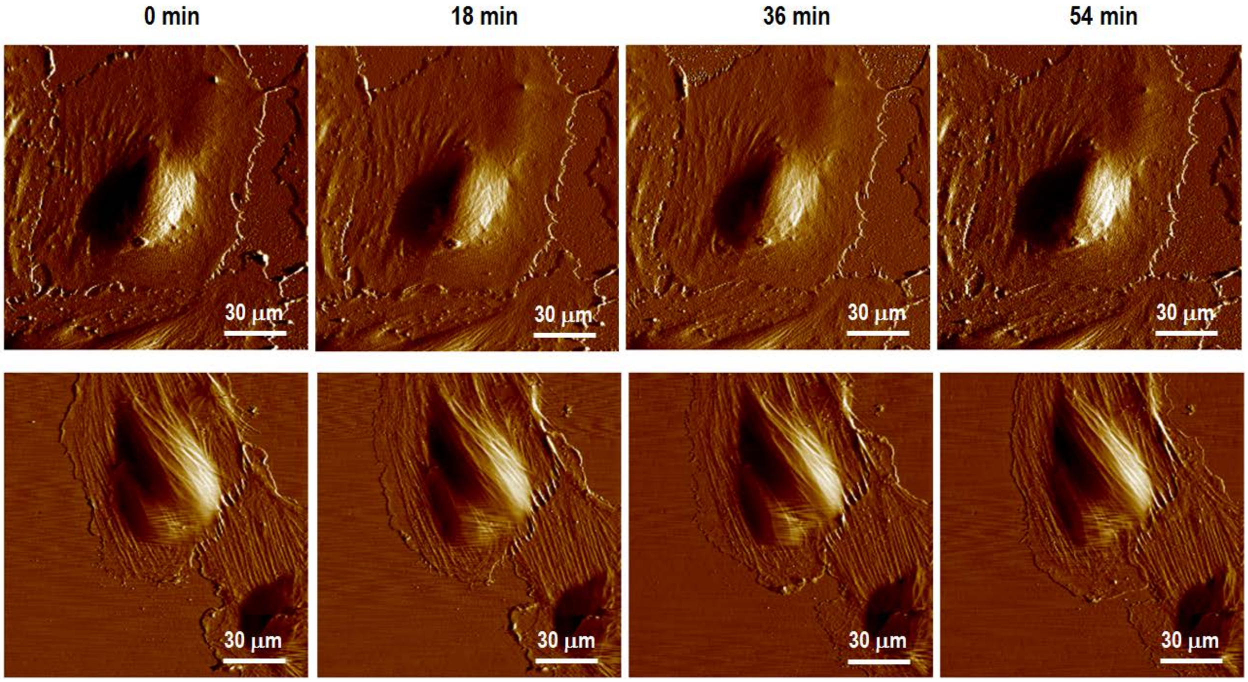

Figure S4. Time-lapse morphology of live human lung endothelial cells without any stimulation

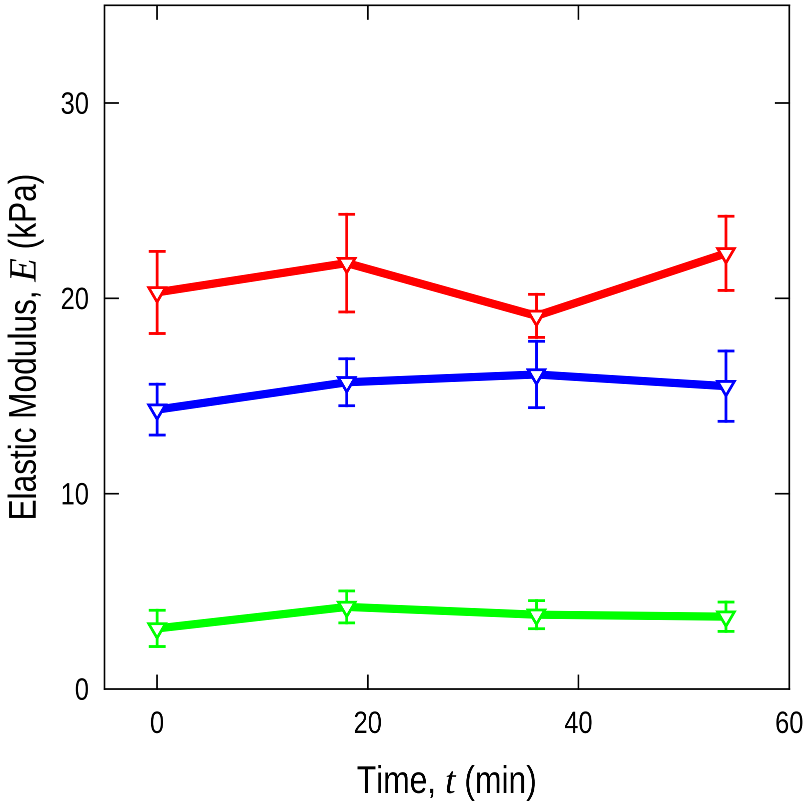

Figure S5. Time-lapse localized elastic modulus on non-stimulated endothelial cells

The mechanical measurements on the non-stimulated cells are also performed as shown in Figure S5 and Table S1. There is no obvious time-lapse tendency seen from the non-stimulated cells. As shown in Figure 4 and Table S2 for endothelial cells treated with S1P at t = 18 min, the elastic modulus at the periphery and cytoplasm are increased after S1P stimulation, due to the actin cytoskeleton is enhanced at the cell edges, seen as membrane ruffles and cortical rings. However, pretreatment with the Abl kinase inhibitor imatinib attenuates S1P-mediated structural changes in human lung EC, reflected by dimishing the increase of elastic modulus along the periphery due to the reduced formation of cortical ring formation. These findings demonstrate that imatinib attenuates multiple cytoskeletal changes associated with S1P-mediated endothelial barrier enhancement and suggest a novel role for Abl kinases in mediating these S1P effects.

Table S1. Time-lapse localized elastic modulus on non-stimulated endothelial cells

Time, t (min)	Periphery, E_1 (kPa)	Nucleus, E_2 (kPa)	Cytoplasm, E_3 (kPa)
0	20.3 ± 2.1	3.1 ± 0.93	14.3 ± 1.3
18	21.8 ± 2.5	4.2 ± 0.82	15.7 ± 1.2
36	19.1 ± 1.1	3.8 ± 0.72	16.1 ± 1.7
54	22.3 ± 1.9	3.7 ± 0.75	15.5 ± 1.8

Table S2. Time-lapse localized elastic modulus on endothelial cells treated with S1P at t = 18 min

Time, t (min)	Periphery, E_1 (kPa)	Nucleus, E_2 (kPa)	Cytoplasm, E_3 (kPa)
0	21.76 ± 1.1	4.23 ± 0.9	15.67 ± 1.8
18	30.89 ± 1.5	5.73 ± 0.9	17.85 ± 1.14
36	32.15 ± 1.68	6.48 ± 1.07	21.54 ± 1.4
54	33.55 ± 1.1	6.5 ± 0.55	22.0 ± 0.8
72	34.0 ± 1.6	6.8 ± 0.75	22.5 ± 0.7
90	34.2 ± 1.3	7.0 ± 1.2	22.9 ± 1.5

Table S3. Time-lapse localized elastic modulus on endothelial cell treated with imatinib (20 μM) at t = 18 min and S1P (20 μM) at t = 72 min

Time, t (min)	Periphery, E_1 (kPa)	Nucleus, E_2 (kPa)	Cytoplasm, E_3 (kPa)
0	21.76 ± 1.1	4.23 ± 0.9	15.67 ± 1.8
18	22.89 ± 1.2	5.0 ± 0.8	16.1 ± 0.95
36	24.15 ± 1.3	5.2 ± 0.9	17.0 ± 1.1
54	24.55 ± 1.1	5.25 ± 0.6	16.5 ± 0.9
72	26.0 ± 1.3	5.3 ± 0.8	17.5 ± 0.9
90	26.5 ± 1.1	5.35 ± 1.1	18.0 ± 1.1

Quantitative analysis on immunofluorescence images

To obtain quantitative analysis on how Abl kinases inhibition with imatinib affects the formation of cortical actin band, image analysis was performed on the immunofluorescence channel using imageJ (6). Each area of interest is considered as a particle to analyze using Log3D plugin (Figure S6A, C and E). Bare outline ranges shown in Figure S6B, D and F are the selected areas used for the quantitative analysis. The quantitative analysis was performed on $n = 20$ images at each condition and the combination effect of imatinib and S1P significantly diminishes the S1P only-induced enhancement on both parameters ($p < 0.005$, determined by two-tailed unpaired Student's t-test).

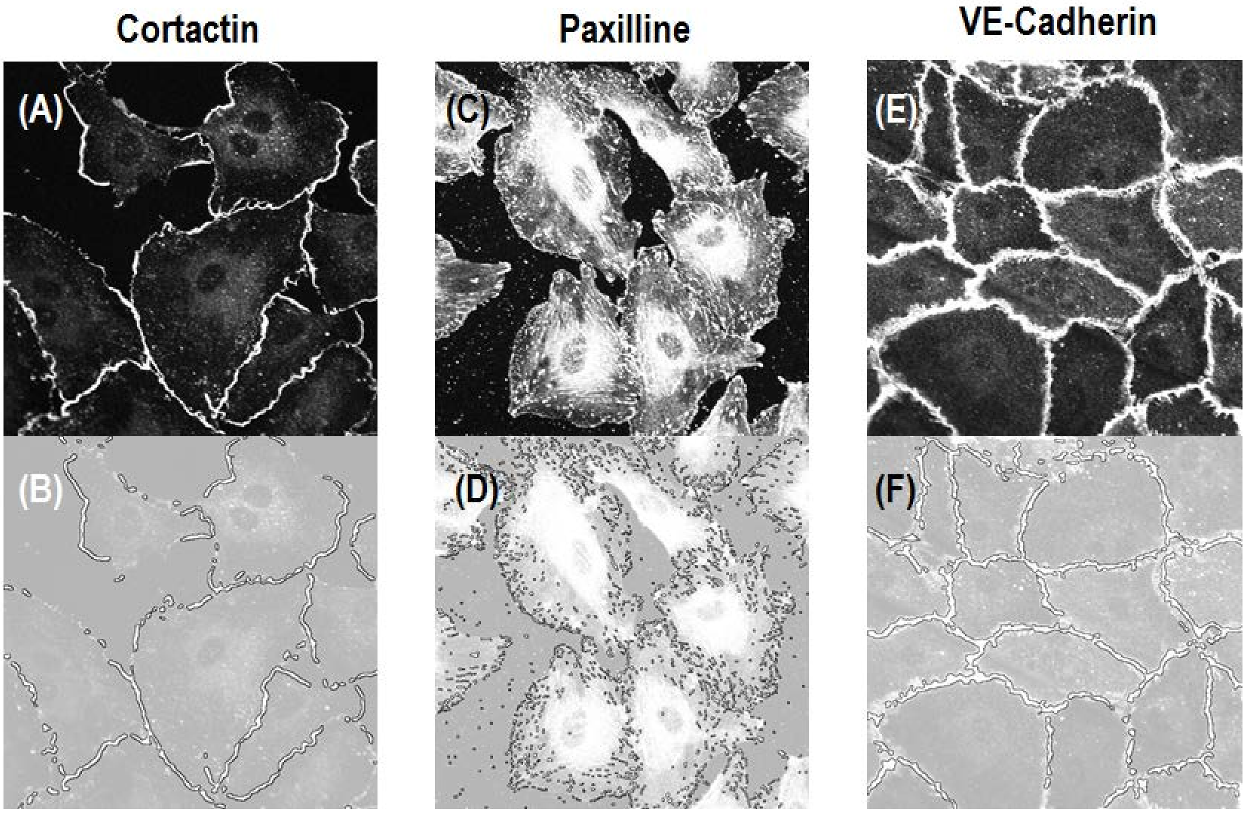

Figure S6. Quantitative analysis on immunofluorescence images of cortactin, paxillin and VE-cadherin (A, C and E) and bare ranges outlined for analyzing proteins of interest (B, D and F)

Reference

1. Sneddon, I.N, *The relation between load and penetration in the axisymmetric boussinesq problem for a punch of arbitrary profile.* International Journal of Engineering Science, 1965. **3**(1): p. 47-57.
2. Carl, P. and Schillers, H., *Elasticity measurement of living cells with an atomic force microscope: data acquisition and processing.* Pflugers Archiv-European Journal of Physiology, 2008. **457**(2): p. 551-559.
3. Iyer, S, Gaikwad, RM, Subba-Rao, V, Woodworth, CD, and Sokolov, Igor, *Atomic force microscopy detects differences in the surface brush of normal and cancerous cells.* Nature nanotechnology, 2009. **4**(6): p. 389-393.
4. Dokukin, Maxim E, Guz, Nataliia V, and Sokolov, Igor, *Quantitative study of the elastic modulus of loosely attached cells in AFM indentation experiments.* Biophysical journal, 2013. **104**(10): p. 2123-2131.
5. Vargas-Pinto, R, Gong, H, Vahabikashi, A, and Johnson, M, *The effect of the endothelial cell cortex on atomic force microscopy measurements.* Biophysical journal, 2013. **105**(2): p. 300-309.
6. Elosegui-Artola, A., Jorge-Penas, A., Moreno-Arotzena, O., Oregi, A., Lasa, M., Garcia-Aznar, J. M., De Juan-Pardo, E. M., and Aldabe, R., *Image Analysis for the Quantitative Comparison of Stress Fibers and Focal Adhesions.* Plos One, 2014. **9**(9).
